# Implantation metastasis from sigmoid colon cancer to rectal anastomosis proved by whole exome sequencing and lineage inference for cancer heterogeneity and evolution analysis: Case report and literature review

**DOI:** 10.3389/fonc.2022.930715

**Published:** 2022-09-20

**Authors:** Guan Yu Yu, Xian Hua Gao, Li Jian Xia, De Bin Sun, Tao Liu, Wei Zhang

**Affiliations:** ^1^ Department of Colorectal Surgery, Changhai Hospital, Naval Medical University, Shanghai, China; ^2^ Hereditary Colorectal Cancer Center and Genetic Block Center of Familial Cancer, Changhai Hospital, Shanghai, China; ^3^ Department of Gastrointestinal Surgery, The First Affiliated Hospital of Shandong First Medical University, Ji'nan, Shandong, China; ^4^ Department of Medicine, Genecast Biotechnology Co. Ltd, Wuxi, China; ^5^ Department of Anorectal Surgery, Zaozhuang Central Hospital, Shandong, China

**Keywords:** implantation metastasis, colorectal cancer, anastomosis, suture line, whole exome sequencing, procedure for prolapse and hemorrhoids

## Abstract

It was estimated that 70% of patients with colorectal cancer were found to have viable exfoliated malignant cells in adjacent intestinal lumen. Exfoliated malignant cells had been reported to implant on raw surfaces, such as polypectomy site, anal fissure, anal fistula, hemorrhoidectomy wound, and anastomotic suture line. Tumors at anastomosis could be classified into four groups: local recurrence, local manifestation of widespread metastasis, metachronous carcinogenesis, and implantation metastasis. However, all of the previous studies only reported the phenomena of implantation metastasis at anastomosis. No study had proved the origin of anastomotic metastasis by genomic analysis. In this study, a 43-year-old woman presented with persistent hematochezia was diagnosed as having severe mixed hemorrhoids. She was treated by procedure for prolapse and hemorrhoids (PPH), without receiving preoperative colonoscopy. Two months later, she was found to have sigmoid colon cancer by colonoscopy due to continuous hematochezia and received radical sigmoidectomy. Postoperative histological examination confirmed the lesion to be a moderately differentiated adenocarcinoma (pT3N1M0). Six months later, she presented with hematochezia again and colonoscopy revealed two tumors at the rectal anastomosis of PPH. Both tumors were confirmed to be moderately differentiated adenocarcinoma without lymph node and distant metastasis and were finally removed by transanal endoscopic microsurgery (TEM). Pathological examination, whole exome sequencing (WES), and Lineage Inference for Cancer Heterogeneity and Evolution (LICHeE) analysis demonstrated that the two tumors at the rectal anastomosis were probably implantation metastases arising from the previous sigmoid colon cancer. This is the first study to prove implantation metastasis from colon cancer to a distal anastomosis by WES and LICHeE analysis. Therefore, it is recommended to rule out colorectal cancer in proximal large bowel before performing surgery with a rectal anastomosis, such as PPH and anterior resection. For patients with a suspected implanted tumor, WES and LICHeE could be used to differentiate implantation metastasis from metachronous carcinogenesis.

## Introduction

Colorectal cancer (CRC) is one of the most common malignancies worldwide. CRC can be spread by direct extension, lymphatic and blood vasculature, and intra-abdominal/transperitoneal spread (1). Implantation metastasis of exfoliated cancer cells on the impaired mucosa of distal bowel had been reported to be another rare way of metastasis. It was estimated that 70% of patients with CRC were found to have viable, exfoliated malignant cells in the proximal and distal lumen adjacent to the tumor (2, 3). Exfoliated malignant cells were reported to implant on raw surfaces, such as polypectomy site (4–6), endoscopic biopsy site (7), wound of anal fissure (8), track of anal fistula (9–11), hemorrhoidectomy scar (1, 12, 13), perianal skin (14, 15), and hook insertion site of Lone Star retractors (16). Implanted metastasis had also been reported in colorectal stapled suture line (17), and it may lead to anastomotic recurrence (18–20). Several studies had reported cases of anastomotic recurrence in patients with CRC treated by stapled anastomosis (21–24). For tumors at anastomosis, they could be classified into four groups: local recurrence, local manifestation of widespread metastasis, metachronous carcinogenesis, and implantation metastasis (25). Possible causes of anastomotic recurrence included positive resection margin, inadequate lymph node dissection, implantation of exfoliated cancer cells (26), germline mutation of susceptibility gene of CRC, and altered biological features of the suture line (25). Anastomotic proliferative instability caused by suture materials (e.g., staples) had been reported to promote the engraftment of exfoliated tumor cells (27, 28). The differential diagnosis of anastomotic tumor is very important, since the treatment plan and prognosis vary greatly (25). However, all of the previous studies only reported the phenomena of implantation metastasis. No study yet provided strong evidence to clarify the origin of metastasis from the molecular point. In theory, genomic analysis and comparative study of the original and anastomotic tumor could help us demonstrate true implantation metastasis (5, 22). This study aims to differentiate implantation metastasis from metachronous carcinogenesis with whole exome sequencing (WES) and Lineage Inference for Cancer Heterogeneity and Evolution (LICHeE) analysis.

## Case presentation

**Figure 1:**
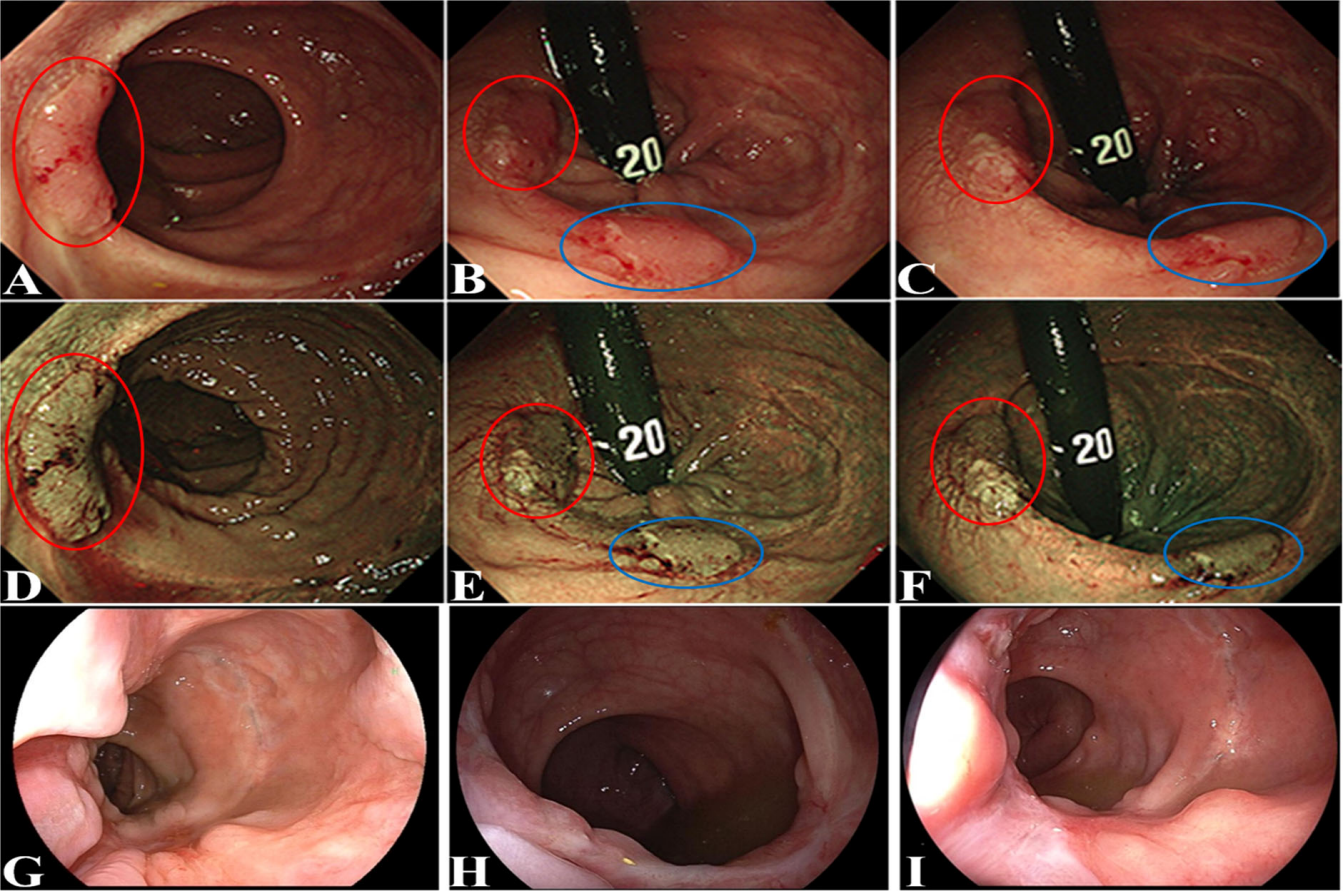
Flexible colonoscopy revealed the two tumors at the rectal anastomosis site of PPH. One tumor was indicated by a red circle, and the other tumor was indicated by a blue circle. **(A–C)**: bright light image; **(D–F)**: narrow band image; follow-up colonoscopy performed at 1 month **(G)**, 3 months **(H)**, and 8 months **(I)** after TEM.

**Figure 2:**
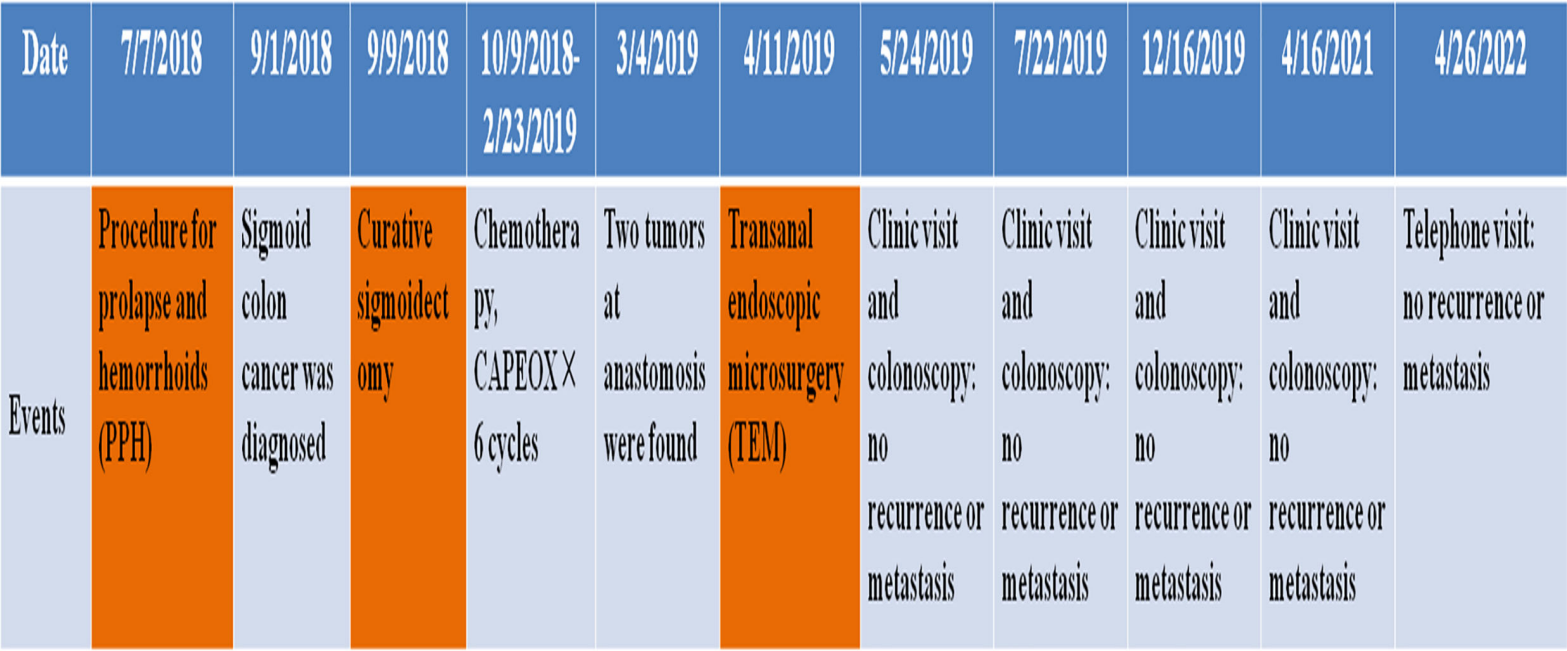
The timeline of important events, treatment, and follow up.

A 43-year-old woman presented with persistent hematochezia. She did not have special history of medical, psychosocial and hereditary disease, relevant past interventions, and family history of cancer. No obvious mass was palpated on digital rectal examination. Preoperative colonoscopy was recommended but refused by the patient. She was diagnosed as having severe mixed hemorrhoids and treated by procedure for prolapse and hemorrhoids (PPH) on 7 July 2018 at a local hospital. Unfortunately, hematochezia continued after PPH, and the patient visited our hospital approximately 2 months after PPH. Colonoscopy (1 September 2018) revealed an ulcerative mass in the sigmoid colon (approximately 20 cm from the anal verge), which was confirmed to be adenocarcinoma on biopsy-based pathological examination. The rectal anastomosis performed during PPH had healed well, and no obvious abnormality was identified at the site of rectal anastomosis under colonoscopy. The patient underwent radical sigmoidectomy on 9 September 2018. Postoperative histological examination confirmed the lesion to be a moderately differentiated adenocarcinoma (pT3N1M0). She underwent 6 cycles of adjuvant chemotherapy (CAPEOX: oxaliplatin 130 mg/m^2^ IV day 1, capecitabine 10,00 mg/m^2^ twice daily PO for 14 days, repeat every 3 weeks) from 9 October 2018 to 23 February 2019. Furthermore, she was followed up regularly at our hospital. Six months after radical excision, she presented with hematochezia again. Flexible colonoscopy (9 March 2019) revealed two tumors at the rectal anastomosis of PPH (**Figure 1**); the sigmoid anastomosis and remaining colorectal mucosa appeared to have normal results. Both tumors at the site of rectal anastomosis were confirmed to be moderately differentiated adenocarcinoma on biopsy-based pathological examination. Lymph node involvement and distant metastasis were excluded by chest CT scan and liver and pelvic contrast MRI. After a careful and thorough discussion between the surgeons and patient, the last two cycles of the planned 8 cycles of chemotherapy were canceled to deal with the latest emerging rectal tumors. The two rectal tumors were removed by transanal endoscopic microsurgery (TEM) on 11 April 2019. Follow-up colonoscopy was performed at 1, 3, and 8 months and 2 years after TEM (**Figures 1G–I** **)**. The patient was last followed up on 26 April 2022 by telephone interview, and she had remained disease-free (without local recurrence or distant metastasis) for 3 years after TEM. The timeline of important events, treatment, and follow up is shown in **Figure 2**.

**Figure 3:**
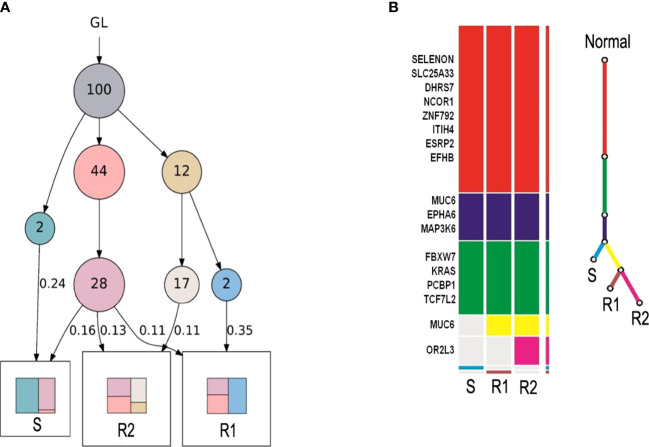
LICHeE analysis was used to assess the diversity using single-nucleotide variants in the three tumor tissues. **(A)** Each circle indicates a clone. The arrow indicates evolutionary direction. The number in the circle indicates the number of variants. GL indicates germline mutations, representing healthy tissues. Colors in squares indicate compositions of clones. Samples sharing common clones in the early stage indicate the presence of metastasis. **(B)** Color bar on the left: each color bar represents a clone, and the length of the color bar represents the number of variants. Color line on the right: the root (red) represents healthy tissues, and the end represents tumor tissues. Tissue with common variants indicates the presence of metastasis.

To explore whether these two rectal tumors (R1 and R2 tumor) were implantation metastases originating from the sigmoid colon carcinoma (S tumor), peripheral blood of the patient and formalin-fixed paraffin-embedded specimens of the three tumors were obtained and analyzed by WES. LICHeE analysis is a well-accepted combinatorial algorithm designed to reconstruct multi-sample cell lineage trees and infer the subclonal composition of the given samples based on variant allele frequencies of single-nucleotide variants (29–31). Therefore, LICHeE analysis was used to assess the diversity using single-nucleotide variants obtained from the three tumor tissues (**Figure 3A**) and showed that the R1 and R2 tumors were probably implantation metastases arising from the **S tumor** (**Figure 3B**).

## Discussion

Implantation metastasis of CRC to a distal anastomosis suture line is a rare situation, which has not yet been proved in the published literature by genomic analysis. To the best of our knowledge, this is the first study to prove the implantation metastasis of CRC to rectal anastomosis by comprehensive analysis, including pathological examination, WES, and LICHeE analysis. Differential diagnosis between implantation metastasis and metachronous carcinogenesis is very important, since treatment plan and prognosis vary greatly. In this case, the mucosa between the rectal anastomosis and sigmoid anastomosis was normal under colonoscopy; postoperative pathological examination showed that the sigmoid colon cancer and the two tumors at rectal anastomosis have similar histology; all of them were moderately differentiated adenocarcinoma. In addition, lymph node and distant metastasis were excluded. These above clinicopathological features indicated that the two anastomotic tumors probably originated from the sigmoid colon cancer. However, all of this evidence is only supportive, not conclusive. The best available method to prove the relationship between two tumors is WES and comparative study with LICHeE analysis, but it is time-consuming and very expensive. For these reasons, none of the published case reports of implantation metastasis had been proved by WES and LICHeE analysis.

It has been demonstrated that viable exfoliated cancer cells presented within the bowel lumen both proximal and distal to the CRC mass (12). Operation manipulation, and the transportation of intestinal content over an ulcerative tumor, may promote cancer cell shedding from the primary tumor and entering the bowel cavity (32). Intact mucosa is highly resistant to tumor cell implantation, and the exfoliated tumor cells cannot implant on normal colorectal mucosa. Instead, they can only implant on open wounds or ulcerated areas (8, 33). Many studies had noticed viable cancer cells in colonic irrigation solution and stapler, and staplers can promote the implantation and growth of viable cancer cells (19). Exfoliated cells may proliferate along the anastomotic site, leading to an implanted tumor. There are two possibilities about the timing of implantation metastasis in this case. First, the implantation metastasis at the anastomosis site may have already occurred shortly after PPH, before sigmoidectomy. The sigmoid colon cancer in this case is an ulcerative mass. Tumor cells may shed from the ulcerative mass, disperse into the lumen, and eventually lead to implantation metastasis at rectal anastomosis. At the time of sigmoidectomy (2 months after PPH), the lesions of implantation metastasis were probably too small to be observed at the well-healed rectal anastomosis under colonoscopy, and they grew larger gradually thereafter. Second, the implantation metastasis may occur at or after sigmoidectomy. Tumor mobilization during sigmoidectomy may cause exfoliation of cancer cells. The rectal anastomoses have probably healed at 2 months after PPH, but the inflammatory response at the anastomosis may also promote cancer cell growth and lead to implantation metastasis at the anastomosis (11).

Due to the rarity of implantation metastasis at the rectal anastomosis, there is no well-established operation. Several operative procedures had been reported in the literature, including local excision, abdominoperineal resection, and anterior resection with anorectal anastomosis (19). In this case, the two anastomotic tumors were proved to be local lesions without lymph node or distant metastasis, so the TEM was selected. At 2 years after TEM, no local recurrence and metastasis were identified, which means that implantation metastases have been cured by TEM. Therefore, TEM and careful monitoring may be adequate for some patients with implantation metastasis at the rectal anastomosis. In order to reduce the incidence of implantation metastasis, the following measures should be considered to control intraluminal dissemination of cancer cells: ligatures of the lumen proximal and distal to the tumor prior to mobilization; thorough irrigation of the bowel lumen with povidone iodine; and washing of the intestinal cavity of distal large bowel with chlorhexidine-cetrimide before cutting the intestine (3, 12, 19, 32). In addition, it should be cautious to perform hemorrhoidectomy or endoscopic excision of colorectal polyps before the removal of CRC, due to the potential increased risk of implantation metastasis to the fresh polypectomy site.

## Conclusion

This is the first study to prove the implantation metastasis of CRC to rectal anastomosis by WES and LICHeE analysis. This case highlights the catastrophic consequence of tumor implantation to a distal rectal anastomosis after radical excision of colon cancer. Therefore, it is recommended to rule out CRC in proximal large bowel before performing surgery with a rectal anastomosis, such as PPH for hemorrhoids and anterior resection for rectal cancer. Once the diagnosis of CRC is confirmed, hemorrhoidectomy (especially PPH) should not be performed until the removal of CRC. Similarly, for patients with a confirmed diagnosis of CRC, a complete colonoscopy is still required to rule out another synchronous CRC in the proximal colon. If the colonoscope cannot pass through the lumen of the tumor site, a careful intraoperative exploration of the unchecked proximal colon is suggested to rule out a second tumor. Otherwise, the exfoliated malignant cells of the proximal tumor may implant at distal anastomosis. For patients with a suspected implanted tumor, WES and LICHeE could be used to differentiate implantation metastasis from metachronous carcinogenesis. Furthermore, TEM could be a reasonable and curative operation for rectal implanted tumor limited within the intestine wall.

## Data availability statement

The original contributions presented in the study are included in the article/supplementary material, further inquiries can be directed to the corresponding author/s.

## Ethics statement

The studies involving human participants were reviewed and approved by the ethics committee of Shanghai Changhai Hospital. The patients/participants provided their written informed consent to participate in this study. Written informed consent was obtained from the patient for the publication of any potentially identifiable images or data included in this article.

## Author contributions

GY and XG contributed equally to this article. GY and XG: material preparation, data collection, and draft preparation. LX and TL: data collection, critical revision, and final approval of the paper DS: data analysis, critical revision, and final approval of the paper. WZ: study conception, design, and final approval of the paper. All authors contributed to the article and approved the submitted version.

## Funding

This study was supported by the National Natural Science Foundation of China (#82072750, #81572332), Shanghai Pujiang Program (#2019PJD052), Natural Science Fund of Shanghai (20ZR1457200), and 234 Subject Climbing Plan of Changhai Hospital (#2020YXK022) Shanghai Sailing Program (21YF1459300), The 71st Batch of China Postdoctoral Science Foundation (48804).

## Conflict of interest

Author DS is employed by the Department of Medicine, Genecast Biotechnology Co. Ltd., Wuxi, China.

The remaining authors declare that the research was conducted in the absence of any commercial or financial relationships that could be construed as a potential conflict of interest.

## Publisher’s note

All claims expressed in this article are solely those of the authors and do not necessarily represent those of their affiliated organizations, or those of the publisher, the editors and the reviewers. Any product that may be evaluated in this article, or claim that may be made by its manufacturer, is not guaranteed or endorsed by the publisher.
